# A unifying theory of synaptic long-term plasticity based on a sparse distribution of synaptic strength

**DOI:** 10.3389/fnsyn.2014.00003

**Published:** 2014-03-04

**Authors:** Daniel Krieg, Jochen Triesch

**Affiliations:** Frankfurt Institute for Advanced Studies, Goethe UniversityFrankfurt, Germany

**Keywords:** synaptic plasticity, sparseness, computational, STDP, metaplasticity

## Abstract

Long-term synaptic plasticity is fundamental to learning and network function. It has been studied under various induction protocols and depends on firing rates, membrane voltage, and precise timing of action potentials. These protocols show different facets of a common underlying mechanism but they are mostly modeled as distinct phenomena. Here, we show that all of these different dependencies can be explained from a single computational principle. The objective is a sparse distribution of excitatory synaptic strength, which may help to reduce metabolic costs associated with synaptic transmission. Based on this objective we derive a stochastic gradient ascent learning rule which is of differential-Hebbian type. It is formulated in biophysical quantities and can be related to current mechanistic theories of synaptic plasticity. The learning rule accounts for experimental findings from all major induction protocols and explains a classic phenomenon of metaplasticity. Furthermore, our model predicts the existence of metaplasticity for spike-timing-dependent plasticity Thus, we provide a theory of long-term synaptic plasticity that unifies different induction protocols and provides a connection between functional and mechanistic levels of description.

## Introduction

Synaptic long-term plasticity, the bidirectional modification of synaptic strength, has a complex dependence on various factors. Among them are direct factors such as correlated pre- and postsynaptic firing rates (Bliss and Lømo, 1973), postsynaptic membrane potential (Artola et al., 1990; Artola and Singer, 1993; Ngezahayo et al., 2000), precise timing of pre- and postsynaptic spikes (Gerstner et al., 1996; Markram, 1997), repetition frequency of such timing (Sjöström et al., 2001), synaptic location (Froemke et al., 2005; Sjöström and Häusser, 2006), as well as indirect (or meta-) factors such as previous postsynaptic activity (Bienenstock et al., 1982; Wang and Wagner, 1999) and initial synaptic strength (Ngezahayo et al., 2000).

Theories and models for many of these factors (especially the timing-dependence) have been studied on different levels of abstractions including biophysical (Lisman, 1989; Artola and Singer, 1993; Shouval et al., 2002), phenomenological (Pfister and Gerstner, 2006; Clopath and Gerstner, 2010; El Boustani et al., 2012), and functional (Toyoizumi et al., 2005; Sprekeler et al., 2007; Pool and Mato, 2011). Nevertheless, synaptic long-term plasticity is induced by a complex molecular machinery. Depending on the experimental protocol we are only probing different facets of this process. But so far, most theories only address a specific induction protocol and none of the existing theories bridges the gap between the biophysical and the functional level.

Here, we present a unifying theory that describes long-term synaptic plasticity from a single objective function based on sparseness. We propose that the primary goal of synaptic long-term plasticity is a sparse distribution of synaptic strength and that synaptic changes induced by STDP and other induction protocols can be understood as a consequence of this objective. The synaptic learning rule resulting from this functional goal is formulated in biophysical quantities and relies only on local quantities. It reproduces results from spike-timing-, rate-, and voltage-dependent induction protocols and even metaplasticity, thus providing a unifying account of synaptic long-term plasticity.

While it has been known for years that the distribution of excitatory synaptic strengths is highly skewed (Song et al., 2005; Loewenstein et al., 2011) and that models of STDP can produce such distributions (van Rossum et al., 2000; Leen and Friel, 2012; Zheng et al., 2013), here we postulate sparseness as the *objective* of long-term plasticity. We argue that this is beneficial because it reduces metabolic costs and increases coding efficiency. Our approach suggests that sparseness may play an even more important role for neural coding than previously thought (Olshausen and Field, 1996; Hromádka et al., 2008).

## Model

This work rests on the hypothesis that synaptic long-term plasticity is actively maximizing the sparseness of the distribution of synaptic efficacies. While this goal appears rather simple, achieving it is not trivial because a single synapse is severely constrained by its computational abilities and what signals are locally available. The aim is a local, computationally simple, and biologically plausible learning rule which explains the experimental data and is consistent with current mechanistic accounts of synaptic plasticity. To derive such a plasticity rule, constraints and approximations have to be considered which the synapse might be using. In the following, the assumptions and the main idea for the derivation is sketched, while more details can be found in the Methods section and the Appendix.

### Proxy and sparseness measure

The goal is a plasticity rule which, when applied locally at every synapse, increases the sparseness of the overall distribution of synaptic efficacies. This requires every single synapse to have a way of estimating this sparseness without having knowledge about the full distribution itself. It needs a proxy to gather information about the strengths of other synapses. The derivations in this work will be built on a simple, but reasonable candidate: the membrane potential *u*. The idea is that the membrane potential can provide the required information throughout the whole dendritic tree. Furthermore, sparseness measures like skewness and excess kurtosis for the distribution of synaptic efficacies can be assumed to be approximately linear in these measures for the membrane potential as argued in the next section.

We assume that the limited computational capacity provided by the biophysical mechanisms in the neuron is not sufficient to directly compute the complex dependence of the membrane potential distribution on the synaptic inputs. A simple approximation in terms of a first order expansion of the voltage dynamics makes the dependence explicit. The time course of the membrane potential is assumed to be linear with additive Gaussian channel noise (Steinmetz et al., 2000) over a short time period δ. Thus, we assume a Wiener process *X*_δ_ with a drift term $\dot{u}$(*t*_0_) and the noise strength σ:

(1) $$u(t_{0}+\delta )\approx u(t_{0})+\dot{u}(t_{0})\delta +\sigma X_{\delta }.$$

The conditional probability density over this time interval is found to be Gaussian as 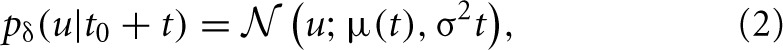 with μ(*t*) = *u*(*t*_0_) + *t*$\dot{u}$(*t*_0_). Apart from voltage-gated ion channels and passive conductances, the velocity of the membrane potential $\dot{u}$ depends linearly on the total synaptic current and thereby on the distribution of synaptic weights.

Next, the measure for the sparseness needs to be specified. Straightforward choices are the statistical measures of skewness *S* or kurtosis *K*, which are frequently used in learning algorithms such as independent component analysis (Hyvärinen and Oja, 2000). They have also been used to derive a BCM-type of learning rule (Blais et al., 1998). In our approach, both measures lead to a very similar learning rule. We will focus on skewness in the following. To calculate these measures the probability density function, or at least its moments, need to be estimated.

### Skewness relation between membrane potential and synaptic efficacies

Given the proxy variable *u*, we will argue that, in a rough approximation, the skewness of the membrane potential is proportional to the skewness of the synaptic efficacies. The objective, thus, reduces to maximizing the skewness of the membrane potential.

Comparing the short duration of the EPSC of about 20–100 ms to the low average firing rate of cortical neurons, we could neglect the finite values of the EPSCs and just describe the synapses as active or inactive. Thus, at every instant in time a small number *n* of all synapses is “active” meaning a presynaptic spike has arrived. The total synaptic current can be compared to the sum of synaptic efficacies as *I*_tot_ ~ ∑_*k* ∈ active_ *w_k_*.

By neglecting correlations between different inputs, the total synaptic current becomes equivalent to the sum of iid samples *w_k_* drawn from the distribution of synaptic efficacies *W*.

For a given state in phase space (*u*, $\dot{u}$), the distribution of the membrane potential depends only on the weights *w_k_* of the “active” inputs due to the first-order expansion in (1). It follows from basic statistics that the skewness of the membrane potential is linear in the skewness of *W*, since the strengths *w_k_* can be regarded as iid samples from *W*. In the long-term average, the skewness values are related as $S_{u}\propto \frac{S_{W}}{\sqrt{n}}$. Here, *n* is the average number of simultaneously active inputs.

Thus, in order to maximize the sparseness of synaptic strength, the aim of the learning rule at each synapse is to maximize the skewness of the membrane potential which is taken as a proxy. It is defined as the third normalized moment of the mean-free membrane potential *û* = *u* − *u*:

(3) $$S_{u}=\frac{\langle {\hat{u}}^{3}\rangle }{{\langle {\hat{u}}^{2}\rangle }^{3/2}}.$$

### Stochastic gradient ascent and moments

The objective function in form of the skewness is maximized via gradient ascent. That means, the derivative of the objective with respect to the synaptic efficacy is used in the learning rule:

(4) $$\dot{w}\propto \frac{\partial S_{u}}{\partial w}$$

The weight follows this gradient and evolves such that the skewness increases.

Using the full expression of the skewness has two implausible requirements: first, the synapse needs to store information about the membrane potential distribution across time and second, it must perform the derivative on this complex expression. A stochastic gradient ascent avoids both problems. Here, the synapse does not estimate the distribution and expectation values of *u* over an extended period of time. Rather, the expectation values are calculated for a very short time interval δ during which we can linearize the dynamics [see Equation (1)].

The weight follows the gradient of a stochastic instantaneous “sample” of the skewness:

(5) $$S_{u}^{*}=\frac{{\langle {\hat{u}}^{3}\rangle }_{\delta }}{{\langle {\hat{u}}^{2}\rangle }_{\delta }^{3/2}}.$$

On a short time scale, the weight will fluctuate seemingly at random while it performs an optimization very local in time. The synapse approximates the complex and unkown skewness landscape and follows the gradient of this approximation. On a longer time scale these flucuations average to an overall change which optimizes the full skewness expression. However, this greedy optimization can potentially get stuck in local optima. Thus, with the introduction of a proxy and the optimization via a stochastic gradient ascent, the original problem of skewness maximization for the weight distribution has been converted from being global in time and space to a learning rule being local in time and space.

The skewness is measured relative to the mean membrane potential *u* which defines the reference point for the distribution and its moments. The requirement for the stochastic gradient ascent to converge is that *u* is not averaged within each “sample” bin. It needs to be estimated over a longer period of time:

(6) $$\bar{u}={\langle u\rangle }_{\tau_{\bar{u}}\gg \delta }.$$

This long-term averaging of *u* is important. We will take *u* to be a low-pass filtered version of the voltage dynamics and compute it via an exponentially weighted average:

(7) $$\frac{\partial \bar{u}}{\partial t}=\frac{u-\bar{u}}{\tau_{\bar{u}}}.$$

To summarize the introduced approximations: the sparseness of the distribution of synaptic efficacies, measured with the normalized higher-order moment of skewness, is estimated using the membrane potential as a proxy. For biological plausibility, the sparseness of the membrane potential is maximized in a computationally simple way by applying a stochastic gradient ascent. The stochastic “samples” are averaged over a very small time interval during which the dynamics of the voltage can be approximated by a first-order expansion. The mean membrane potential, however, is averaged over an extended time period.

Calculating the “sample” skewness from (5) amounts to evaluating the moments of the proxy variable over δ. The moments of the mean-free membrane potential *û* = *u* − *u* can be done analytically due to the Wiener approximation from (2). The *n*-th moment is found as: 
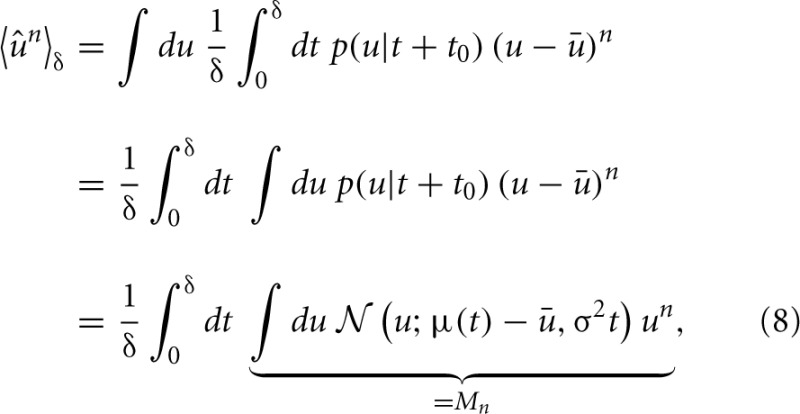
 where *M_n_* is just the n-th raw moment of a Gaussian, since the order of integration can be interchanged according to Fubini's theorem. The moments 〈*û*^*n*^〉_δ_ follow as

(9) $${\langle {\hat{u}}^{2}\rangle }_{\delta }={\hat{u}}^{2}+\frac{1}{2}\left(\sigma^{2}+2\hat{u}\dot{u}\right)\delta +\frac{1}{3}{\dot{u}}^{2}\delta^{2}$$

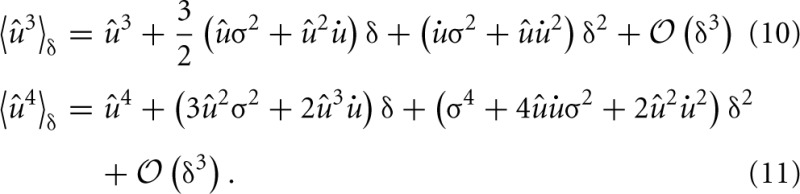


### Learning rule

The synaptic efficacy (or weight) *w* is a single number characterizing the strength of the synapse. It is the result of a combination of different factors, e.g., released neurotransmitter, number of receptors, and single channel conductance of the receptors. Thus, the weight is a function of different factors *x_i_*: *w* = *w*(*x*_1_, …, *x_m_*). These are the final variables inducing plasticity which, in order to maximize the skewness, follow the differential equation

(12) $${\dot{x}}_{i}:=\eta_{i}\frac{\partial S_{u}^{*}}{\partial x_{i}}$$

(13) $$\;=\eta_{i}\frac{\partial w}{\partial x_{i}}\frac{\partial \dot{u}}{\partial w}\frac{\partial S_{u}^{*}}{\partial \dot{u}},$$

with learning rates η_*i*_. The gradient decomposes into three terms since the skewness only depends on $\dot{u}$ which depends on *w* which in turn depends on *x_i_*.

#### The first term

$\frac{\partial w}{\partial x_{i}}$ depends on how the weight and its regulating mechanisms/variables are modeled. We will restrict ourselves to the common postsynaptic factors of number and single channel conductance of the receptors. For a glutamatergic synapse, there are two main types *R* of receptors (NMDA and AMPA) each having an average maximal single channel conductance *g_R_* and a total number *N_R_*. The synaptic current of a given receptor type *R* is given by

(14) $$I_{R}(t)=\underset{w_{R}}{\underset{︸}{N_{R}g_{R}}}\;\underset{I_{R}^{\left(0\right)}(t)}{\underset{︸}{g_{0}^{R}(t)\left(E_{R}-u\right)}},$$

with the resulting weight *w_R_*, the normalized current of a single receptor *I*^(0)^_*R*_(*t*) and the reversal potential *E_R_*.

Each quantity will be optimized simultaneously with individual learning rates:

(15) $${\dot{N}}_{R}:=\eta_{N}\frac{\partial w_{R}}{\partial N_{R}}\frac{\partial \dot{u}}{\partial w_{R}}\frac{\partial S_{u}^{*}}{\partial \dot{u}}=\eta_{N}g_{R}\;\Omega$$

(16) $${\dot{g}}_{R}:=\eta_{g}\frac{\partial w_{R}}{\partial g_{R}}\frac{\partial \dot{u}}{\partial w_{R}}\frac{\partial S_{u}^{*}}{\partial \dot{u}}=\eta_{g}N_{R}\;\Omega$$

The common expression $\Omega =\frac{\partial \dot{u}}{\partial w}\frac{\partial S_{u}^{*}}{\partial \dot{u}}$ does not depend on *N* and *g*. The coupling of the differential equations depends on the learning rates η_*g*_ and η_*N*_. They determine the behavior of the weight change

(17) $${\dot{w}}_{R}=g_{R}{\dot{N}}_{R}+N_{R}{\dot{g}}_{R}.$$

Here, the learning rates in all simulations were set such that the weight change was multiplicative:

(18) $${\dot{w}}_{R}=2\sqrt{\eta_{g}\eta_{N}}w_{R}\Omega .$$

An additive learning rule can be obtained with a different setting of the learning rates but leads to qualitatively similar results. The derivations for the dependence of *w_R_* on the learning rates is described in the Methods: Derivation of the learning rule in Appendix.

#### The second term

$\frac{\partial \dot{u}}{\partial w}$ is simply linear in the input current for a standard conductance-based neuron model. The derivative of the membrane potential depends linearly on the synaptic current *I*_syn_(*t*) = ∑*_R_w_R_I^(0)^_R_*(*t*) and thus

(19) $$\frac{\partial \dot{u}}{\partial w_{R}}=\frac{I_{R}^{\left(0\right)}(t)}{C},$$

where *I*^(0)^_*R*_(*t*) is the single channel current through a receptor of type *R* and *C* is the membrane capacitance. While the current through a single channel *I*^(0)^_*R*_ is a quantity that can not be determined by a synapse, the overall plasticity rule depends on the total synaptic current for the multiplicative case from (18).

#### The third term

Establishes the connection to the sparseness measure and is therefore the one which is fundamental to the approach. Using the identities from (9) to (11), we take the derivative of S^*^_u_ with respect to $\dot{u}$ and expand in orders of the small time interval δ: 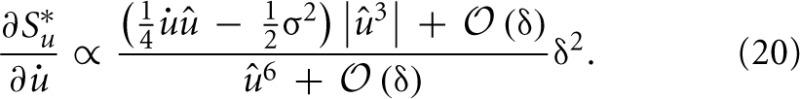


All terms in S^*^_u_ which are of order δ^0^ do not depend on $\dot{u}$ and the terms of order δ^1^ cancel out exactly. Thus, we are left only with terms of order δ^2^ or higher. The overall factor δ^2^ can be absorbed into the learning rate and will be neglected. For *û* → 0 the higher-order terms in δ can not be neglected. Instead of using the full gradient expression, we introduced a simple regularization parameter γ. It is a constant phenomenological parameter which represents the higher-order terms and thereby prevents the expression from diverging:

(21) $$\frac{\partial S_{u}^{*}}{\partial \dot{u}}\approx \frac{1}{4}\frac{\left(\dot{u}\hat{u}-2\sigma^{2}\right)\left|{\hat{u}}^{3}\right|}{{\hat{u}}^{6}+\gamma^{6}}\delta^{2}$$

(22) $$\;\overset{\hat{u}\gg \gamma }{\to }\;\frac{1}{4}\frac{\dot{u}\hat{u}-2\sigma^{2}}{\left|{\hat{u}}^{3}\right|}\delta^{2}.$$

### Differential Hebbian learning

The final plasticity rule with all three terms combined, for skewness as the objective function, is

(23) $${\dot{w}}_{R}:=\eta \frac{1}{4}\frac{I_{R}(t)}{C}\;\frac{\left(\dot{u}\hat{u}-2\sigma^{2}\right)\left|{\hat{u}}^{3}\right|}{{\hat{u}}^{6}+\gamma^{6}}.$$

Using kurtosis as the sparseness measure results in the similar expression

(24) $${\dot{w}}_{R}:=\eta \frac{2}{3}\frac{I_{R}(t)}{C}\;\frac{\left(\dot{u}\hat{u}-1.5\sigma^{2}\right){\hat{u}}^{5}}{{\hat{u}}^{8}+\gamma^{8}}.$$

Here, all remaining constant factors and parameters were combined into one parameter: the learning rate $\eta =2\sqrt{\eta_{g}\eta_{N}}\delta^{2}$ now has the dimension of seconds.

The differential equations exhibit a strongly non-linear dependence on the mean-free membrane potential *û*. In both cases the functional form of this dependence is similar. If *û* is very small, the noise parameter σ dominates the numerator and the expression is negative. For a positive $\dot{u}$, the numerator will be zero for some intermediate value of *û* and become positive for larger values. The whole expression, dominated by the denominator, approaches zero again for sufficiently large depolarizations. This dependence of the plasticity on the membrane potential resembles the experimental findings on voltage-dependent plasticity as we will show in the next section.

The other important functional dependence is on the correlation between the time derivative of the membrane potential $\dot{u}$ and the synaptic input current *I_R_*. Neglecting the small noise parameter σ, the plasticity rule is proportional to their product as

(25) $${\dot{w}}_{R}\propto \dot{u}I_{R}.$$

Thus, it belongs to the class of so-called differential Hebbian rules where the weight change depends on the correlation between presynaptic activity and the derivative of postsynaptic activity (Kosko, 1986).

## Results

### Weight distribution and skewness

**Figure 1:**
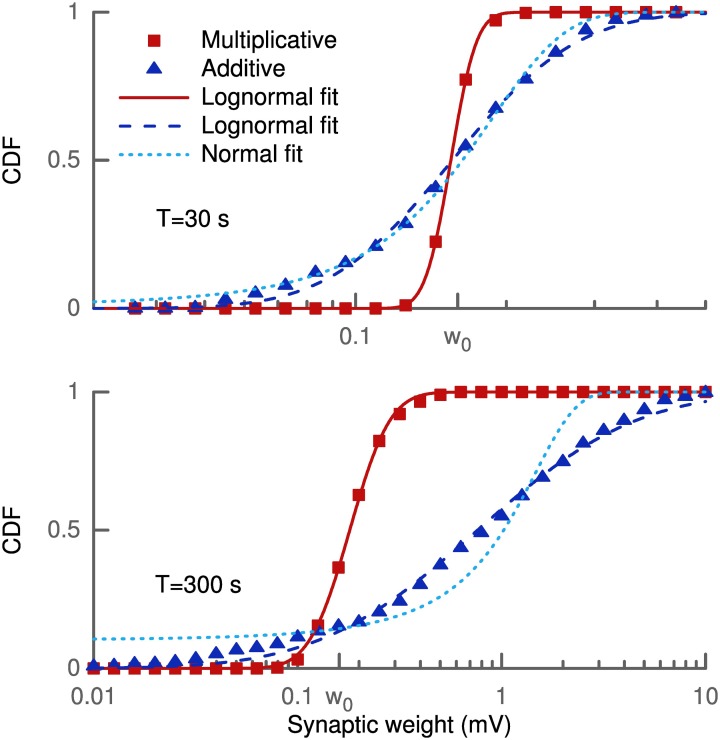
**Cumulative density function of synaptic weights for additive and multiplicative learning rule**. The weights for the 100 synapses are initialized at *w*_0_ = 0.16 mV and shown for two different simulation durations (upper and lower panel). The cumulative densities for multiplicative and additive weight changes are fitted with a lognormal (straight and dashed line). Additionally, a Gaussian (dotted line) is fitted to the additive weight changes.

The learning rule was derived from the goal of maximizing skewness. Therefore, we assessed the effect of the learning rule on the distribution of synaptic weights and its skewness. 100 synapses located at 200 μm from the soma received independent presynaptic Poisson spike trains at 3 Hz. The weight distribution was estimated over the population of 100 synapses for several trials. In each trial every synaptic efficacy was initially set to *w*_0_ = 0.16 mV and new random spike trains were sampled over the given duration (see Figure 1). Since we did not employ any saturation effects or hard bounds, some synapses grew strong enough to trigger a postsynaptic spike. This spike strongly biased the measured strength of these synapses (see Methods). We, therefore, excluded all synapses with a strength larger than 10 mV from our skewness analysis to remove these non-linear effects.

The simulations were done with our standard multiplicative weight change and also for the additive weight change (see Methods and Appendix). The reason for this was that any multiplicative weight change following some distribution ultimately leads to a lognormal distribution according to the central limit theorem and Gibrat's law. And such a lognormal distribution will always exhibit a positive skewness. Thus, the effect of the learning rule might not be distinguishable from random weight changes. On the other hand, for an additive random weight change following some distribution the synaptic efficacies would become Gaussian due to the central limit theorem. A lognormal distribution even for additive changes shows that the objective of an increased skewness is actually achieved by the learning rule.

Figure 1 shows the cumulative density function of synaptic weights for two different simulation durations (*T* = 30 s, 300 s) in log-scale. The cumulative density function, rather than the probability density function, was chosen to eliminate statistical problems with the choice of the histogram bin sizes. The weights from the multiplicative learning rule are well fitted by a lognormal distribution for both durations as expected. But also the additive learning rule leads to a cumulative density function which follows a lognormal distribution. For the short simulation of 30 s (upper panel) the Gaussian fit is only slightly worse in terms of the root mean square of the residuals (RMSR) (*RMSR*_lognormal_ = 0.0209, *RMSR*_Gaussian_ = 0.0238), but it becomes worse as the weight change progresses. The weights at 300 s show a slight deviation from the lognormal cumulative density function at low values while the Gaussian does not fit the data very well (*RMSR*_lognormal_ = 0.0227, *RMSR*_Gaussian_ = 0.0814).

**Figure 2:**
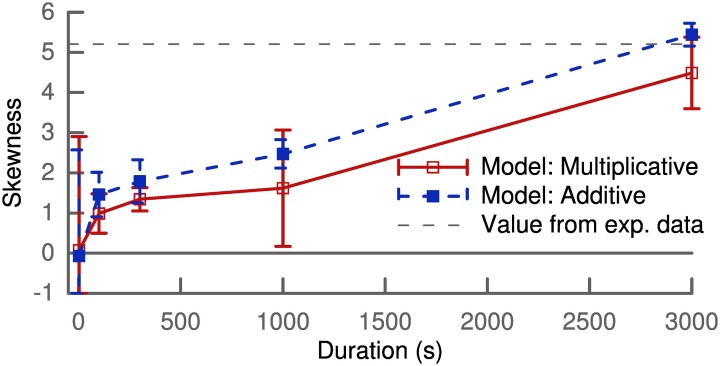
**Skewness of the synaptic weight distribution over simulation duration**. The horizontal dashed lines correspond to the value calculated from a lognormal fit to experimental data (Song et al., 2005). Results shown are calculated for one population of 100 synapses with the mean and variance of skewness averaged over several trials.

Figure 2 shows the skewness of the weight distribution as a function of simulation duration. Both types of learning rules show an increase of skewness from the initial delta peak, which is not skewed, to values of around 4 after 5 min of simulation. These values are comparable to the value of 5.2 extracted from a lognormal fit to experimental data (Song et al., 2005).

### STDP: spike pairings

**Figure 3:**
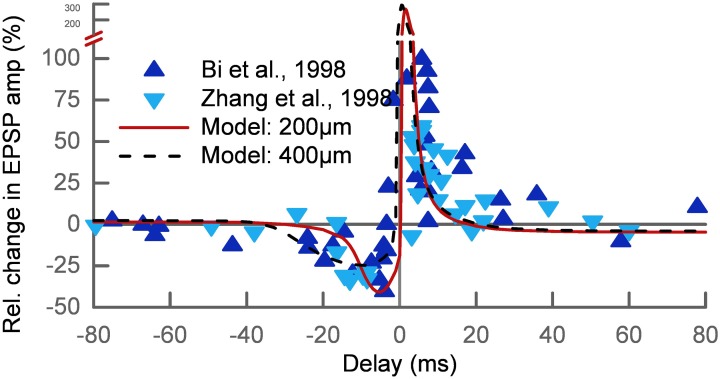
**Relative change of the synaptic strength in an STDP pairing protocol**. The experimental data (Bi and Poo, 1998; Zhang et al., 1998) (triangles) show LTD for post-pre pairs and LTP for pre-post pairs with delays up to 30–40 ms. The results of our model are shown for two different synaptic locations on the apical dendrite: 200 μm (solid line) and 400 μm (dashed line) from the soma. The timescale of the LTD window increases with distance while the LTP window shows no dependence on the location.

We tested if our approach can explain the basic phenomenon of STDP. The main protocol for studying STDP is by pairing pre- and postsynaptic action potentials at different time delays Δ at low repetition frequency. For a negative delay the postsynaptic spike precedes the presynaptic spike (post-pre) and for a positive delay the order is reversed (pre-post). We simulated 7 spike pairings at 1 Hz for two different synapses at 200 μm (*w*_AMPA_ = 200 pS) and 400 μm (*w*_AMPA_ = 250 pS) distance from the soma. The results were in good agreement with the experimental data (Bi and Poo, 1998; Zhang et al., 1998) apart from the LTP at very short delays, which is predicted too strong (Figure 3).

It should be noted that the 7 spike pairings we have used are not comparable to the more than 50 pairings which are usually required in experiments to achieve these amounts of plasticity. One possible explanation is the learning rate which we kept constant for all simulations in this manuscript in order to make consistent comparisons. But one could consider different learning rates for different cell types which would lead to different number of spike pairings required. However, the question how many pairings are required is not unimportant. On the one hand, using more spike pairings would lead to much more plasticity in our simulations, which would be problematic especially for very short delays. On the other hand, it is also questionable that plasticity can be generated with such few pairings applied. For these cases to be handled realistically we would need more detailed mechanisms for the expression and maintenance of plasticity which we did not include so far.

Interestingly, the STDP window depended on the synaptic location. This is related to the broadening of the backpropagating action potential (bAP) along the dendritic tree. The change in the timescale of the bAP decay affects the interaction of post-pre pairs. The width of the LTD window in our model is therefore increasing with distance from the soma, while the LTP window is relatively independent of the synaptic location. We show this by way of example for two synapses at 200 μm and 400 μm (Figure 3). This feature of our model was also found experimentally (Froemke et al., 2005).

### STDP: frequency dependence

**Figure 4:**
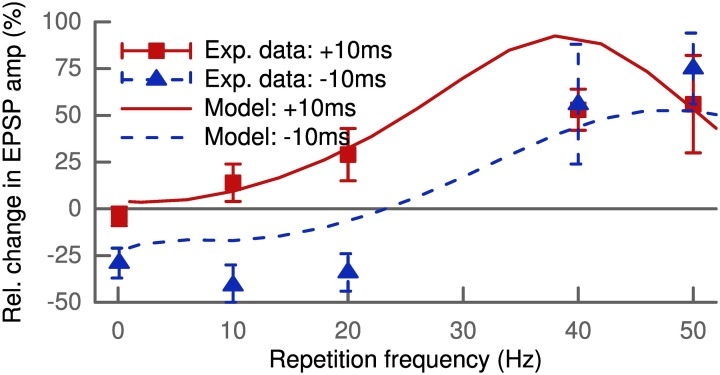
**Increasing the repetition frequency of the STDP pairing leads to non-linear effects**. Delays were fixed at +10 ms (squares and solid line) or −10 ms (triangles and dashed line). The experimental data (Sjöström et al., 2001) show LTP for pre-post at low frequencies up to 20 Hz which increases with frequency, while post-pre pairing leads to LTD with roughly constant amplitude. At frequencies above 30 Hz LTP for pre-post starts to saturate, but post-pre pairings exhibit a crossover from LTD to LTP. At 50 Hz both result in strong LTP, since pre-post and post-pre protocols become equivalent for Δ = ± 10 ms.

The standard STDP protocol consists of spike pairings repeated at low frequencies. Increasing this repetition frequency leads to interactions between spikes of different pairs. The LTP and LTD of these pairings do not add linearly as shown by experimental findings (Sjöström et al., 2001). Figure 4 shows our simulation results for 100 pairings at positive (+10ms) and negative (−10ms) delays as a function of the repetition frequency compared to the experimental data. The modeled synapse was located proximally at 150 μm and only contained NMDA receptors (*w*_AMPA_ = 0 pS). This is comparable to the experimental conditions where the measured excitatory postsynaptic potential only showed one slow component with decay times of about 50 ms (Sjöström et al., 2001). At low frequencies below 5 Hz, the model predicted LTD for negative pairings but hardly any LTP for positive pairings.

The vanishing LTP at low pairing frequencies might seem to be in conflict to the results of Figure 3, but we retrieved the missing LTP with more distal synapses containing AMPA receptors. Interestingly, this is in line with results from Pawlak and Kerr (2008) showing a non-zero LTP for very low pairing frequencies (<0.1 Hz). Their results were based on synapses containing mainly AMPA receptors as demonstrated by the AMPA/kainate blocker CNQX, as compared to the setup by Sjöström et al. (2001) with a dominant NMDA contribution to the EPSC.

LTP for positive pairings increased with frequency and the LTD for negative pairings was converted to LTP for frequencies above 30 Hz. At 50 Hz, where both protocols became equivalent, robust LTP was predicted by the model matching the experimental data (Sjöström et al., 2001) (Figure 4). Thus, our model can account for the non-linear integration of LTP and LTD when looking beyond isolated spike pairs.

**Figure 5:**
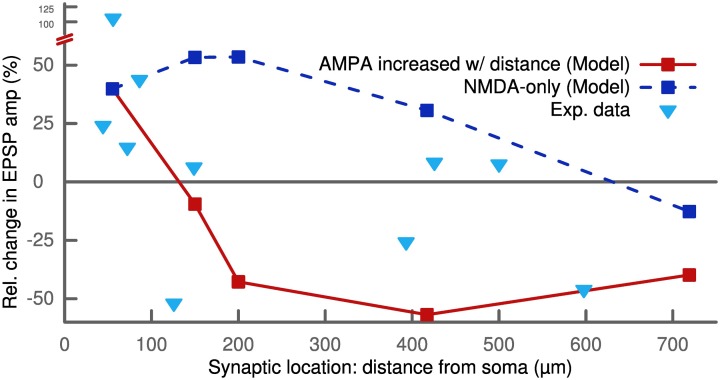
**STDP pairings with a delay of +10 ms at 50 Hz for different synaptic locations**. Proximal synapses (<200 μm) show strong LTP. In the case of no AMPA receptors (dashed line), the potentiation decreases with distance starting at 200 μm and eventually converts to LTD above 700 μm. Increasing the number of AMPA receptors with distance (solid line) abolishes the potentiation and converts it to LTD. Proximal synapses with a distance to the soma smaller than 200 μm were assumed to be dominated by NMDA receptors and did not contain any AMPA receptors (see Methods). The experimental data (triangles) show a similar switch from LTP to LTD as a function of distance (Sjöström and Häusser, 2006).

Like for the standard STDP protocol, we observed a dependence on the synaptic location for this frequency-dependent STDP protocol. The robust potentiation at 50 Hz for ±10 ms pairings was only measured at proximal synapses. It converted to a slight depression for very distal locations more than 700 μm from the soma. We observed this LTP-LTD switch for NMDA-only synapses as well as for synapses with a distance-dependent increase in AMPA receptors (Figure 5). These results of our model are qualitatively similar to experimental findings (Sjöström and Häusser, 2006).

### Rate-dependent plasticity and metaplasticity

**Figure 6:**
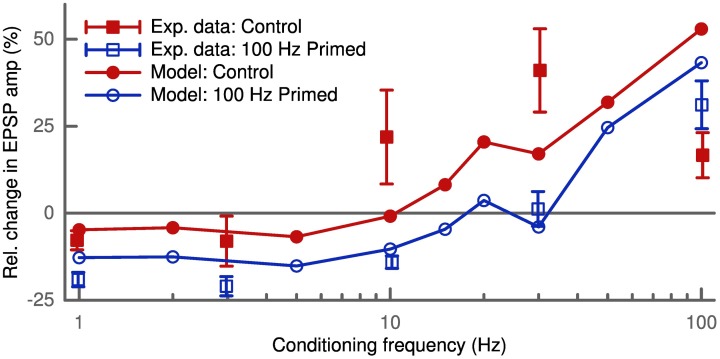
**Activity-dependent metaplasticity in accordance with the BCM theory**. The experimental data (Wang and Wagner, 1999) in the control conditions (filled squares) reveal slight LTD at low conditioning frequency (<5 Hz) and strong LTP above. After priming the postsynaptic cell with a 100 Hz stimulus (empty squares) the LTD window becomes larger (up to 30 Hz) and stronger (amplitude doubles). In the model the control case (filled circles) corresponds to an average postsynaptic activity history of 10 Hz, while in the priming case (empty circles) the neuron was stimulated with 100 Hz for 3 s before the conditioning. Upon priming the model displays the same leftward shift of LTD/LTP crossover point and an increase in LTD amplitude.

Depression and potentiation can also be induced without a controlled timing of pre- and postsynaptic spikes. We simulated this rate-dependent plasticity by independent pre- and postsynaptic Poisson spike trains lasting 30 s. The simulated synapse was the same as in the STDP pairing protocol (200 μm distance from the soma with *w*_AMPA_ = 200 pS). The presynaptic frequency was fixed at 10 Hz and we probed the induced plasticity as a function of the postsynaptic conditioning frequency. Our model predicted LTD at low conditioning frequencies and LTP above a given frequency threshold (Figure 6). Furthermore, increasing the presynaptic frequency increased the amplitude of the observed change in synaptic strength (not shown). These results agree with the predictions of the BCM theory (Bienenstock et al., 1982) and also fit the data of its experimental verification (Wang and Wagner, 1999).

Another prediction of the BCM theory is the so-called metaplasticity, where the history of postsynaptic activity influences the observed plasticity. To simulate an increased postsynaptic activity the cell is primed with a 100 Hz stimulus (Wang and Wagner, 1999) which we applied for 3 s prior to the conditioning. The high postsynaptic activity increased the average membrane potential *u* at the synapse due to the bAP. This led to a larger amount of LTD (Figure 6) as found experimentally (Wang and Wagner, 1999). This homeostatic effect is of heterosynaptic nature, since the bAP influences *u* at all synapses.

**Figure 7:**
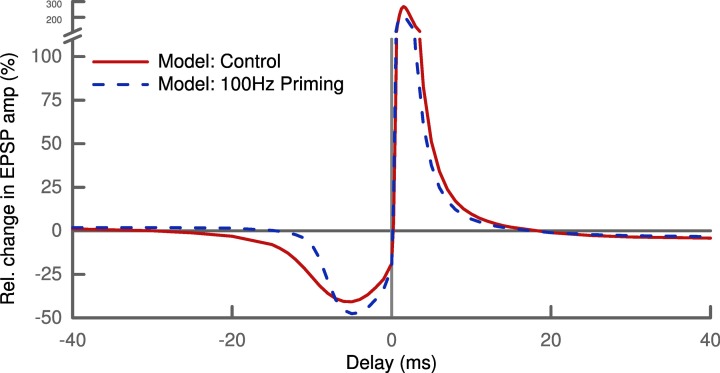
**Shape of the STDP pairing window depends on history of activity**. Priming the postsynaptic cell with a 100 Hz stimulus (dashed line) alters the amplitude of LTD and LTP slightly. Most notably, the priming decreases the width of the LTD window by a factor of two: LTD is not induced for post-pre pairs with delays greater than ~10 ms compared ~20 ms under control conditions (solid line).

So far, metaplasticity has only been studied for the above mentioned rate-dependent protocol. The level of abstraction used in our model does not allow us to make novel predictions in a quantitative way. But combining STDP with a priming protocol for metaplasticity results in a clear qualitative prediction. Our model predicts that the width of the LTD window in an STDP protocol will be smaller if it is preceded by strong priming (Figure 7). This prediction is a fundamental consequence of our learning rule given the influence of the priming, namely raising the mean membrane potential *u*. It is also not depending on the choice of skewness as the sparseness measure, but will be valid also for kurtosis or any other higher moment.

### Voltage-dependent plasticity

**Figure 8:**
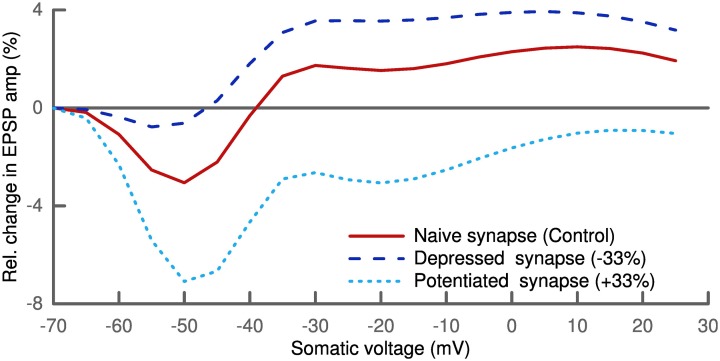
**Plasticity depends on postsynaptic depolarization**. Simulation of a distal synapse (700 μm from soma) with only NMDA receptors. Pairing presynaptic spikes with postsynaptic voltage clamping at the soma leads to no change for slight depolarization, LTD for intermediate and LTP for strong depolarization (solid line). We scaled the parameter σ linearly with the initial synaptic strength for the depressed and potentiated synapses (see Text). Depressed synapses (dashed line) have a smaller LTD window and potentiated synapses (dotted line) a larger one.

Finally, we tested if our model can also explain results from an induction protocol based on clamping postsynaptic membrane voltage. To this end, we paired a presynaptic stimulation with postsynaptic depolarization. We clamped the soma of the postsynaptic cell at different voltages. A distal synapse (700 μm) with only NMDA receptors was stimulated 100 times at a frequency of 2 Hz following the protocol from Ngezahayo et al. (2000). No change was induced when clamping the membrane voltage around the resting potential, while low depolarization led to LTD and higher depolarization resulted in LTP (Figure 8).

The general trend of our results is in line with experimental findings (Artola et al., 1990; Feldman, 2000; Ngezahayo et al., 2000). But the amount of plasticity was very sensitive to the synaptic location. In the current formulation of our model, plasticity can only be generated for synapses which are either far from the clamping position and/or have a spine with a small isolating neck. There, the influence of the voltage clamp is weaker and $\dot{u}$ is not absolutely zero but can slightly fluctuate. Also the effect of AMPA receptors in the synapse was quite pronounced. Increasing the number of AMPA receptors led to more LTP (not shown).

In these voltage clamp simulations we also looked at the influence of the phenomenological parameter σ which we had taken as fixed so far. σ was introduced to account for the influence of noise of the voltage dynamics on its sparseness. This noise is partially originating from the stochasticity of ion channels and synaptic receptors. We therefore scaled σ linearly with the synaptic strength. Interestingly, this homosynaptic scaling of σ had a homeostatic effect. The LTD window of the depressed synapse was decreased, while it increased for the potentiated synapse (Figure 8). A similar shift in the voltage-dependent plasticity was observed experimentally and also shown to be of homosynaptic nature (Ngezahayo et al., 2000).

## Discussion

The phenomenon of synaptic long-term plasticity is driven by a complex mechanism and the diverse experimental induction protocols are only probing different aspects of this process. We showed that induction of spike-timing-, rate-, and voltage-dependent plasticity as well as metaplasticity all follow from the single computational idea of a sparse distribution of synaptic strengths. Our approach has an intrinsic dependence on the synaptic location which agrees with the experimental findings. We therefore propose the maximization of sparseness as a central goal of synaptic plasticity. Our model also leads to a novel prediction of metaplasticity for STDP.

We provide a two-fold unification compared to existing models of synaptic plasticity. Detailed models (Lisman, 1989; Artola and Singer, 1993; Shouval et al., 2002) addressing the biophysical mechanisms can not make conclusions regarding functional goals or consequences. Previous functional models (Toyoizumi et al., 2005; Sprekeler et al., 2007; Pool and Mato, 2011), however, can not be connected to the biophysical mechanisms, since their learning rule is formulated in abstract ways. Also they have only addressed one specific induction protocol, namely STDP for spike pairs. In contrast, our functional model explains the findings from different induction protocols as emerging from one learning rule and it provides a connection to the underlying biophysics by proposing a concrete, biologically plausible learning rule. Our approach bears some similiarity to the independently developed model by Yger and Harris (2013) which also follows the objective of increasing skewness of the membrane potential, however, without reference to the skewness of the weight distribution. While their focus is more on unsupervised learning in a neural network, our focus lies with a biologically tractable learning rule and its effects in a fully morphological neuron. In contrast to the additionally required firing rate constraint by Yger and Harris (2013), our learning rule intrinsically contains a homeostatic metaplasticity to regulate the activity.

### Weight distribution and dendritic properties

We have shown that the learning rule is capable of increasing skewness and can even reach values which are comparable to experiments (Figure 1). However, it is not directly clear where the skewness would saturate. On the one hand, we would require saturation mechanisms for the synaptic weight to prevent some weights from becoming too strong as already explained in the results section. On the other hand, we have used a very simplified setting, with only 100 synapses at equal distance from the soma, just to verify the increase in skewness. In order to actually assess the distribution in a realistic scenario, one would need to have many more synapses distributed along the dendritic tree with random initial strength. Also the input would need to be structured instead of random and could be provided within a network simulation. Ultimately, the setup would depend on the neuron type and brain region one would like to simulate. Assessing such scenarios is beyond the scope of this paper and it is not clear what the actual distribution of synaptic strength would look like. However, we suspect that the skewness would be increased by our learning rule irrespective of the exact scenario.

If the increase in skewness would be a global one or if the optimization would be carried out locally in each branch of the dendritic tree, is an interesting question. This would depend on the geometry and the type of the dendrite, i.e., active or passive. For instance, considering active dendrites (London and Häusser, 2005) would affect the distance-dependent differences in synaptic weight: it improves the transmission of the bAP and can also boost the input of distal synapses. Furthermore, with the generation of local or even global dendritic spikes the plasticity is far less dependent on somatic activity and the activity in one branch of the dendrite can influence the plasticity in connected branches.

### Plasticity protocols

A direct consequence of our differential-Hebbian learning rule is the asymmetric shape of the STDP window. Under conditions of an STDP protocol the time derivative of the membrane potential $\dot{u}$ will mainly depend on the bAP from the soma. Thus, plasticity is driven by an interaction of bAP and excitatory postsynaptic current (Markram, 1997). The location dependence of the STDP window in our model is due to the broadening of the bAP along the dendritic tree. For a pre-post pairing the synaptic current mainly coincides with the rise of the bAP where $\dot{u}$ is positive, while in the post-pre case it only overlaps with the decay where $\dot{u}$ is negative. Such differential-Hebbian learning rules for STDP have already been proposed by Saudargiene et al. (2004). Their model was motivated by reinforcement learning algorithms (Kolodziejski et al., 2009), but they did not reproduce other plasticity phenomena than STDP pairing. A purely phenomenological model by Albers et al. (2013), also based on a differential-hebbian learning rule, is able to fit a range of experimental results like triplets and bursting. Our approach provides a functional justification for the use of differential Hebbian rules in STDP and reinforcement learning, since this type of learning arises naturally from the computational goal of sparse synaptic strength.

An interesting point to speculate about are the observed anti-Hebbian STDP windows (Fino et al., 2005; Letzkus et al., 2006) where LTP is induced with post-pre spikes and LTD with pre-post spikes. An obvious idea would be to propose a minimization of the sparseness, instead of the maximization, which would introduce a minus sign in the overall learning and, thereby, identically reverse the induced plasticity. This would raise the question why some neurons would try to optimize the opposite objective function. However, minimizing the sparseness might not be so strange as it first seems. It would lead to a distribution where most of the synapses are stronger and only a few would be weak. These weak connections can be neglected leaving many synapses with a very similar strength. Together with the homeostatic metaplasticity regulating the average strength, the cell would be driven by the equally weighted inputs of many synapses. In contrast, a cell maximizing the sparseness is driven by only a few very strong inputs.

Previous models have proposed a specific triplet interaction for STDP (Pfister and Gerstner, 2006) to explain the results for the different repetition frequencies (Figure 4). We do not need any additional mechanism in our model to account for these findings. The robust LTP at high frequencies is due to different timescales of LTP and LTD in our model. Induction of LTP is short but strong due to the rising phase of the bAP, while induction of LTD is weaker but develops over a longer time. Thus, LTD needs more time to build up but its induction is cut off at high frequencies by the bAP of the next pairing repetition. So the contributions for potentiation will dominate the depression and effectively result in LTP. The vanishing of LTP at low frequencies arises due to the kinetics of the NMDA receptors. For a pre-post pairing the synapse is near the resting potential on the arrival of the presynaptic spike. Then the NMDA channels are not fully relieved from their Mg^2+^-block at the bAP arrival and the synaptic current is low. At increasing frequencies the bAP from the previous pairing will provide increasing depolarization. This will gradually relieve the Mg^2+^-block and lead to stronger synaptic currents. For a post-pre pairing on the other hand, the cell is still depolarized from the bAP. Then the NMDA channel is already unblocked at low frequencies.

The concept of metaplasticity from the BCM theory (Bienenstock et al., 1982) has a direct analog in our model and does not have to be put in explicitly. For BCM, the firing rate threshold regulating the LTD-LTP crossover is a function of the recent postsynaptic activation in order to maintain a homeostatic balance. In our learning rule, the average membrane potential *u* naturally accounts for this regulation.

The induction of potentiation and depression fits qualitatively with the results from voltage clamp experiments. But the plasticity in our learning rule is linear in $\dot{u}$ which is zero at the location of the clamp. Only synapses which are sufficiently far, where the clamp effect is weaker, can experience a visible amount of plasticity. We hope that this shortcoming of our model in the current formulation can be resolved by extending it with the calcium concentration as a proxy for the distribution of synaptic strength (see Future work).

### Sparseness objective

We have postulated that the goal of synaptic plasticity is the maximization of sparseness of the synaptic weights. There exist many different ideas regarding the advantages of sparseness and sparse coding [see Olshausen and Field (2004) for a review]. Maybe the most appealing interpretation is in terms of energy efficiency. The brain needs to perform its computations with a limited energy budget. On the level of a single neuron it tries to maximize the information transmission for some average firing rate (Attwell and Laughlin, 2001). Thus, sparse coding can be interpreted as a mechanism for energy-efficient coding given that the generation of action potentials is metabolically costly. But the postsynaptic effects of a presynaptic action potential also induce metabolic costs (Attwell and Laughlin, 2001). An excitatory postsynaptic current raises the postsynaptic membrane potential which subsequently has to be brought back to the resting state by restoring ionic concentrations. Given a sparse distribution of synaptic strength, most synapses induce an excitatory postsynaptic potential (EPSP) with low amplitude and low energy consumption, while few synapses induce an EPSP with a high amplitude and high energy consumption. The energy for synaptic transmission will be mainly spent in the few strong synapses, but these have a high probability of inducing a postsynaptic action potential. Thus, the objective of a sparse synaptic strength leads to an energy-efficient synaptic signaling which may be an evolutionary relevant factor.

A second relevant view on the benefits of a sparse synaptic strength is the need for tuning and input specificity. For a neuron to transmit relevant information, it should be tuned to specific inputs and avoid an unspecific Gaussian distributed input. But, according to the central limit theorem, a sum of *n* independent random variables always approaches a Gaussian regardless of their distribution as *n* becomes large. Interestingly, a lognormal weight distribution slows down the convergence toward the Gaussian due to its large skewness (see Note: Effect of sparseness on the central limit theorem in Appendix).

There are many different possible mechanisms that could achieve our computational goal of sparseness. Our model is a result of the optimization via a stochastic gradient ascent, the specific choice of the sparseness measure, and the use of the membrane potential as a proxy for the synaptic weight distribution. The stochastic gradient ascent is necessary to keep the problem computationally simple. For the sparseness measure we have chosen skewness as a higher-order statistical moment. Similarly, one could apply kurtosis or any higher standardized cumulant which would result in largely similar learning rule. An interesting difference between the cumulants is the dependence on the mean-free potential *û*. A learning rule based on skewness (or any odd cumulant) will be negative if *û* is negative, while for kurtosis (or any even cumulant) it does not depend on the sign of *û*. Thus, in the context of metaplasticity, a learning rule based on kurtosis does not lead to stronger depression after a 100 Hz priming. These higher-order moments are usually considered unstable since they can be very sensitive to outliers. We believe, however, that this is not a problem in our approach since the neuron is a bounded dynamical system. The voltage ‘samples’ driving the synaptic changes are continuously distributed and have no outliers.

### Biological realization and future work

We have discussed the computational and algorithmic level of our approach. But our learning rule can also be related to mechanistic theories of synaptic plasticity. Its non-linear dependence on the membrane potential fits with the Artola-Bröcher-Singer (ABS) theory (Artola and Singer, 1993) and is quite comparable to the common U-shaped Ω function characterizing the dependence of synaptic changes on the intracellular calcium concentration [Ca^2+^]_*i*_ (Shouval et al., 2002). There, the voltage dependence of synaptic plasticity has consistently been connected to the surge of [Ca^2+^]_*i*_. In this so-called calcium control hypothesis (Lisman, 1989) it is suggested that different levels of intracellular calcium have opposing influence on the Ca^2+^/calmodulin-dependent protein kinase II (CaMKII). This affects synaptic strength, since CaMKII regulates the phosphorylation level of AMPA receptors which determines channel conductance.

Relating the membrane potential in our learning rule with the calcium concentration also fits with the question how the synapse should estimate $\dot{u}$. It was hypothesized that the calcium-binding protein calmodulin, due to its kinetics, could serve as differentiator of the intracellular calcium concentration (Rao and Sejnowski, 2001). However, an implementation of our learning rule might not necessarily employ a continuous time derivative at every moment in time. A simple estimate of the correlations between the synaptic current and the time derivative of the voltage (or calcium concentration) could be sufficient. In this respect, it was shown that the amplitude of synaptic change in an STDP protocol is strongly correlated with the initial rate of increase of the intracellular calcium concentration over the stimulation period (Aihara et al., 2007).

The strong dependence of our learning rule on the membrane potential is due to the choice of *u* as a proxy to estimate the synaptic weight distribution. It was chosen since its dependence on the presynaptic input and the synaptic efficacies are easy to formulate. Given the important role of calcium in mechanistic models, an important extension to our approach would be to take the intracellular calcium concentration as a proxy. Formulating the objective in this quantity would allow for a very direct connection to the established mechanistic theory of the calcium control hypothesis. The derivation of a learning rule in this case would involve the dependencies of the calcium concentration on presynaptic input, membrane potential, and release from intracellular stores.

With the introduction of a calcium proxy, we expect new effects to be introduced in the results since the non-homogenity of the cytoplasmic calcium could affect the learning rule. For example, considering calcium nanodomains we would expect that the calcium signal would provide information about a much smaller neighborhood of synapses instead. The skewness would then potentially be optimizied for different regions individually instead of for the whole dendrite. However, the main term of the learning rule would depend on the change of the calcium concentration (currently change of the membrane potential), so the effects would not depend so much on the calcium distribution itself but rather on the spatial transmission of changes in this distribution.

With this extension, also the dependence on NMDA and AMPA receptor strength could be formulated more realistically. So far we assumed that the weight change of each receptor type only depends on its own synaptic current. But synaptic plasticity is calcium-dependent and thus mainly mediated by NMDA receptors. Normal AMPA receptors only indirectly affect the calcium concentration due to voltage-dependent calcium channels and the voltage-dependent magnesium block of NMDA receptors. But also the role of calcium-permeable AMPA receptors is just emerging (Man, 2011). All of these dependencies can be incorporated into our approach by extending it with a calcium concentration proxy.

A second important step toward a biologically realistic model is to take into account the actual mechanisms which contribute to the weight change. Our approach is mainly focused on the induction of plasticity and for its expression we have used the simplifying assumption that the number and single-channel conductances of the NMDA- and AMPA-type glutamate receptors are independently adapted as continuous variables. Incorporating realisitic expression, saturation, and maintenance of plasticity into our approach could be done with an explicit model of the discrete conductance/phosphorylation states, their transitions and a model of receptor insertion and removal. These mechansims could potentially account for the discrepancies in our simulations of the STDP pairing protocol. There, the plasticity for short delays grew too strong (requiring saturation) and the number of applied pairings is usually not sufficient to produce long-term plasticity in experiments (requiring some kind of threshold for expression or maintenance).

Also spine neck geometry can influence the results of our learning rule. A strengthened synapse would not only have a large EPSP amplitude at the soma, but also locally in the spine head. This leads to a stronger potentiation since the synaptic current correlates more strongly with the local EPSP. Such a positive feedback could be avoided by regulating the spine neck resistance to achieve a “standardized” EPSP in the spine head (Gulledge et al., 2012).

Finally, it has been shown recently that the metaplasticity threshold is actually independent of postsynaptic action potentials but rather depends on calcium (Hulme et al., 2012). Since our model in the current formulation intrinsically accounts for BCM-like metaplasticity as a function of the mean membrane potential, we expect that this influence of calcium be accounted for when using the calcium proxy extension.

### Conclusions

In the light of our findings, we propose that synaptic plasticity is arising from the computational goal of sparse signaling. David Marr proposed to distinguish three levels of theoretical analysis: computational goal, algorithm, and implementation (Marr, 2010). Our proposal of a sparse synaptic strength (computational goal) leads to a differential-Hebbian learning rule (algorithm) which can be mapped onto the biological mechanisms (implementation), thereby, covering all three levels of analysis. The principle of sparseness would then be even more fundamental to neuronal computation than previously assumed. Given the interpretation in terms of energy efficiency it can be considered a fundamental factor that has shaped the mechanisms of neuronal plasticity during nervous system evolution.

## Methods

We used full morphological simulations in NEURON (Carnevale and Hines, 2006). All simulations were done with a layer 5 pyramidal neuron (Mainen et al., 1995) provided on ModelDB (Hines et al., 2004) (accession number 8210).

The plasticity rule depends on several parameters falling into two categories: The phenomenological model parameters, like the learning rate, were fixed for all experiments. The values are listed in Table 1.

**Table 1:** **The phenomenological model parameters**.

**Description**	**Symbol**	**Value**
Learning rate	η	1.5s
Regularization for *u*	γ	10mV
Noise level	σ^2^	0.036mV^2^ms^−1^
Time constant for *u*	τ_*u*_	30s

The physiological parameters comprise the morphology of the dendritic tree, the synaptic location on this tree, the spine geometry, the initial strength of the synapse, and the contributions of the different receptors types (AMPA and NMDA) as well as their gating kinetics. Those parameters are also relevant in the experimental preparations and can vary between different cell types, brain area, etc.

The dependence of the resulting plasticity on the exact morphology of the tree is beyond the scope of this work. And although the spine geometry is found to depend on the dendritic location (Jones and Powell, 1969; Berard et al., 1981) and influence plasticity (Yuste and Bonhoeffer, 2001; Gulledge et al., 2012), it was also taken to be fixed. The spine consisted of a neck (1 μm long, 0.1 μm thick) and the head (0.6 μm long, 0.3 μm thick) as used by Koch and Poggio (1983).

The time course of the synaptic conductance of a single AMPA/NMDA receptor was modeled by the sum of three exponentials *ĝ*(*t*) with a normalized maximum: one exponential for the rising phase (time constant τ_*r*_), two for the decaying phase (time constants τ_*d*_1__, τ_*d*_2__), and their relative strength λ. The parameter values are listed in Table 2.

(26) $$\;g_{0}^{\text{AMPA}}(t)={\hat{g}}_{\text{AMPA}}(t)$$

(27) $$g_{0}^{\text{NMDA}}(t,u)={\hat{g}}_{\text{NMDA}}(t)\;{\left(1+\frac{{\left[{\text{Mg}}^{2+}\right]}_{o}}{\beta }\exp\left[-\alpha u\right]\right)}^{-1}$$

The additional term accounts for the voltage-dependent magnesium block of the NMDA receptor (Gabbiani et al., 1994). The postsynaptic action potentials were elicited by current injection of 2 mA for 3 ms at the soma.

**Table 2:** **The parameters for the conductance time course *ĝ(t)* of AMPA and NMDA**.

**Parameter**	**AMPA**	**NMDA**
	**(Spruston et al., 1995)**	**(Kinney et al., 1994)**
τ_*r*_	0.55ms	4.05ms
τ_*d*_1__	2.0ms	27.6ms
τ_*d*_1__	8.0ms	147.4ms
λ	0.8	0.5
*N*	2.00	1.36
$\hat{g}(t)=N(\lambda \exp\left[-t/\tau_{d_{1}}\right]+\left(1-\lambda \right)\exp\left[-t/\tau_{d_{2}}\right]-\exp\left[-t/\tau_{r}\right])$		

### Weight and weight change

The total synaptic conductance of a synapse was a weighted sum of both receptor types:

(28) $$g_{\text{total}}(t)=w_{\text{AMPA}}g_{0}^{\text{AMPA}}(t)+w_{\text{NMDA}}g_{0}^{\text{NMDA}}(t)$$

Both weights *w*_R_ independently followed the differential Equation (23) and were changed continuously. The strength of a synapse was measured as the EPSP peak amplitude at the soma. The increase/decrease of synaptic strength was calculated as the relative change of this peak amplitude.

The initial value of the NMDA weight was taken to be independent of the distance to soma with *w*_NMDA_ = 500 pS. The influence of the AMPA current on the plasticity is usually small, since it is mainly calcium dependent. But due to the currently simpler version of our model (membrane potential is used as a proxy quantity instead of calcium concentration), the effect of the AMPA current was too strong and *w*_AMPA_ was adjusted to fit the experimental data. Simulated synapses with a distance to soma smaller than 200 μm did not contain any AMPA receptors. For all other synapses, *w*_AMPA_ was increased with the distance to soma (Andrasfalvy and Magee, 2001) and the resulting total synaptic strength was on the order of 0.1 − 0.2 mV (Magee and Cook, 2000). The actual weights are mentioned in the results section.

## Author contributions

Daniel Krieg designed and performed research, analyzed data and wrote the paper; Jochen Triesch designed research and wrote the paper.

### Conflict of interest statement

The authors declare that the research was conducted in the absence of any commercial or financial relationships that could be construed as a potential conflict of interest.

## Appendix

### Methods: derivation of the learning rule

#### Probability distribution

Sparseness is a function of the moments of a probability distribution. To calculate these moments (or expectation values) over a time interval [*t_0_*, *t*_0_ + *T*] of duration *T*, we need the probability *p*_*T*_(*u* = *u*_0_) that the membrane potential *u* takes a particular value *u*_0_. This is just the time integral over the conditional probability *p*(*u*|*t*) times the “prior” probability *p*(*t*) (which is uniform):

(A1) $$\begin{array}{l} p_{T}(u)=\int_{t_{0}}^{t_{0}+T}dt\;p(u|t)p(t)\\ \;=\frac{1}{T}\int_{t_{0}}^{t_{0}+T}dt\;p(u|t)\\ \;=\frac{1}{T}\int_{0}^{T}dt\;p(u|t+t_{0}) \end{array}$$

In the deterministic case, we can predict *u*(*t*) given initial conditions. Therefore, *p*(*u*|*t* + *t*_0_) = δ(*u* − *u*(*t* + *t*_0_)).

In a real neuron, we can not predict *u*(*t*) for all times, but only for a very short time period. Therefore, we use a stochastic gradient approach. In a stochastic scheme with discrete time one substitutes the required expectation value by a sample. For the continuous time dynamical system of a neuron we take a sample by calculating the probability distribution in a future infinitesimal time interval [*t*_0_, *t*_0_ + δ]. Thereby, the membrane potential can be approximated with first-order dynamics. Assuming an additional iid Gaussian noise (which accounts for synaptic input and the noise of ion channels) leads us to a Wiener process including drift: *u*(*t* + *t*_0_) ≈ μ(*t*) + σ *X_t_* with μ(*t*) = *u*(*t*_0_) + *t*$\dot{u}$(*t*_0_). Thus, the conditional probability will be  In the noiseless limit of σ → 0 we recover the delta distribution.

#### Sparseness measures

We can consider different measures for sparseness. We investigate skewness *S* and kurtosis excess *K* as sparseness measure to be maximized:

(A3) $$\;S=\frac{\mu_{3}}{\sigma^{3}}=\frac{\langle {\hat{u}}^{3}\rangle }{{\langle {\hat{u}}^{2}\rangle }^{1.5}}$$

(A4) $$K=\frac{\mu_{4}}{\sigma^{4}}-3=\frac{\langle {\hat{u}}^{4}\rangle }{{\langle {\hat{u}}^{2}\rangle }^{2}}-3$$

To compute these measures (and their derivatives) we calculate the expectation values of the mean-free membrane potential *û* = *u* − *u* with our stochastic approximation:  where *M_n_* is just the n-th raw moment of a Gaussian

(A6) $$M_{2}={\bar{\mu }}^{2}+\sigma^{2}t$$

(A7) $$M_{3}={\bar{\mu }}^{3}+3\bar{\mu }\sigma^{2}t$$

(A8) $$M_{4}={\bar{\mu }}^{4}+6{\bar{\mu }}^{2}\sigma^{2}t+3\sigma^{4}t^{2}$$

with μ = μ(*t*) − *u*. The integral can now be calculated analytically and we are able to compute the derivatives as:

#### Mean membrane potential

For *û* → 0 one cannot neglect the higher-order terms in δ. Instead of using the full gradient expression for our learning rule, we introduce a simple regularization parameter. γ is a constant phenomenological parameter which represents the higher-order terms and thereby prevents the expression from diverging:

(A11) $$\frac{\partial S}{\partial \dot{u}}\approx \frac{\left(\frac{1}{4}\dot{u}\hat{u}-\frac{1}{2}\sigma^{2}\right)\left|{\hat{u}}^{3}\right|}{{\hat{u}}^{6}+\gamma^{6}}$$

(A12) $$\frac{\partial K}{\partial \dot{u}}\approx \frac{\left(\frac{2}{3}\dot{u}\hat{u}-\sigma^{2}\right){\hat{u}}^{5}}{{\hat{u}}^{8}+\gamma^{8}}$$

Sparseness is measured with respect to the mean of the probability distribution. In our case, it depends on the average membrane potential *u*. Due to the use of the stochastic gradient approach the maximization of the sparseness is achieved as a long-term average. Thus, the mean potential *u* should be basically a low-pass filtered version of the voltage dynamics. It will be varying much more slowly than the instantaneous potential and its derivative. We compute it as an exponentially weighted average: $\frac{\partial \bar{u}}{\partial t}=\frac{u-\bar{u}}{\tau_{\bar{u}}}$.

#### Weight dependence

We use standard neuronal dynamics

$$C\dot{u}=\sum_{\text{ion}}g_{\text{ion}}\left(E_{\text{ion}}-u\right)+\sum_{R}I_{R}(t).$$

The synaptic current of a given receptor type *R* is given by

$$I_{R}(t)=\underset{w_{R}}{\underset{︸}{N_{R}g_{R}}}\underset{=I_{R}^{\left(0\right)}(t)}{\underset{︸}{g_{0}^{R}(t)\left(E_{\text{syn}}^{R}-u\right)}}$$

with the normalized current of a single receptor *I*^(0)^_*R*_(*t*), its maximal conductance *g_R_*, and the total number of receptors *N_R_*.

We optimized both *N* and *g* simultaneously with individual learning rates:

(A13) $$\dot{N}=\eta_{N}\frac{\partial w}{\partial N}\frac{\partial \dot{u}}{\partial w}\frac{\partial S}{\partial \dot{u}}=\eta_{N}g\Omega$$

(A14) $$\dot{g}=\eta_{g}\frac{\partial w}{\partial g}\frac{\partial \dot{u}}{\partial w}\frac{\partial S}{\partial \dot{u}}=\eta_{g}N\Omega$$

Here, $\Omega =\frac{\partial \dot{u}}{\partial w}\frac{\partial S}{\partial \dot{u}}$ does not depend on *N* and *g*. Thus, we have two coupled differential equations. One can decouple them as

(A15) $${\dot{X}}_{1,2}=\lambda_{1,2}X_{1,2}\Omega$$

by introducing $X_{1,2}=\frac{g}{\sqrt{\eta_{g}}}\pm \frac{N}{\sqrt{\eta_{N}}}$ and $\lambda_{1,2}=\pm \sqrt{\eta_{g}\eta_{N}}$. Observing that $\frac{\partial \left(X_{1}\cdot X_{2}\right)}{\partial t}\equiv 0$ it follows that *N* and *g* are constrained on a hyperbola:

$$r^{2}:=\frac{g^{2}}{\eta_{g}}-\frac{N^{2}}{\eta_{N}}\equiv \text{const.}$$

With this constraint we can decouple the initial differential equations as

(A16) $$\dot{N}=\lambda \Omega \sqrt{N^{2}+\eta_{N}r^{2}}$$

(A17) $$\dot{g}=\lambda \Omega \sqrt{g^{2}-\eta_{g}r^{2}}$$

with $\lambda =\lambda_{1}=\sqrt{\eta_{g}\eta_{N}}$. The overall synaptic strength *w* evolves as

(A18) $$\dot{w}=g\dot{N}+N\dot{g}=\lambda \left(\sqrt{w^{2}+\eta_{N}g^{2}r^{2}}+\sqrt{w^{2}-\eta_{g}N^{2}r^{2}}\right)\Omega .$$

The simulation results in this paper were obtained with *r*^2^ = 0. For this case the hyperbola degenerates to a linear relationship between *N* and *g* and the learning rule will be purely multiplicative

$$\dot{w}=2\lambda w\Omega .$$

In the other extreme cases of *r*^2^ → ±∞ we only change one variable and keep the other fixed. This leads to a purely additive learning rule. For example keeping *g* fixed (η_*g*_ = 0) and only updating *N* will lead to

$$\dot{w}=\eta_{N}g^{2}\Omega .$$

For finite values of *r*^2^ the learning rule will be multiplicative for large *w* and gradually become additive for smaller *w*.

### Note: effect of sparseness on the central limit theorem

The central limit theorem states that the distribution of the sample mean of *n* i.i.d. random variables converges to a normal distribution as *n* goes to infinity:  with *E*(*X*) = μ and *E*[(*X* − μ)^2^] = Var(*X*) = σ^2^.

The Barry-Esseen theorem attempts to quantify the rate of convergence in the central limit theorem. It provides an upper bound on the absolute difference between Φ (the cdf of the standard normal distribution) and *F*_*n*_ (the cdf of the normalized sample mean of the i.i.d random variables). It states that for all *x* and *n* there exists a positive constant *C* such that

$$\left|F_{n}(x)-\Phi (x)\right|\leq \frac{C\rho }{\sigma^{3}\sqrt{n}}$$

with the third absolute moment *E*[|*X* − μ|^3^] = ρ < ∞.

Let us interpret the inverse of the upper bound as a convergence rate

$$k:=\frac{\sigma^{3}\sqrt{n}}{C\rho }.$$

Given a fixed number of variables, the rate can be minimized by maximizing the ratio $\frac{\rho }{\sigma^{3}}$ or a lower bound of it. Since ρ is the third absolute moment, a trivial lower bound for this ratio is always the skewness *S* which is the third central moment divided by σ^3^, i.e., *S* < $\frac{\rho }{\sigma^{3}}$. But interestingly, for the log-normal distribution [which synaptic strength is observed to follow (Song et al., 2005; Loewenstein et al., 2011)] one also finds that $\frac{\rho }{\sigma^{3}}$ < *S*(1 + α) where α ∝ $\frac{1}{\sigma^{3}}$ for σ → ∞. That means, *S* < $\frac{\rho }{\sigma^{3}}$ < *S*(1 + α) and the lower bound will become tight for sufficiently large σ. Specifically, for a log-normal fit to experimental data (Song et al., 2005) (*X* ~ ln (−0.702,0.9355^2^)) the bound is already very close with α < 0.05. Thus, maximizing the skewness of synaptic strength will minimize the convergence rate *k*. The total synaptic current will only slowly converge to a Gaussian distribution and remain sparse even for a large number of inputs.
